# Metagenomic and Metatranscriptomic Study of Microbial Metal Resistance in an Acidic Pit Lake

**DOI:** 10.3390/microorganisms8091350

**Published:** 2020-09-04

**Authors:** Diana Ayala-Muñoz, William D. Burgos, Javier Sánchez-España, Estelle Couradeau, Carmen Falagán, Jennifer L. Macalady

**Affiliations:** 1 Department of Civil and Environmental Engineering, The Pennsylvania State University, 212 Sackett Building, University Park, PA 16802, USA; wdb3@psu.edu; 2 Geochemistry and Sustainable Mining Unit, Instituto Geológico y Minero de España (IGME), Calera 1, Tres Cantos, 28760 Madrid, Spain; j.sanchez@igme.es; 3 Department of Ecosystem Science and Management, The Pennsylvania State University, 450 ASI, University Park, PA 16802, USA; estelle@psu.edu; 4 Environment & Sustainability Institute and Camborne School of Mines, University of Exeter, Penryn Campus, Penryn TR10 9FE, UK; c.falagan@exeter.ac.uk; 5 Department of Geosciences, The Pennsylvania State University, 211 Deike Building, University Park, PA 16802, USA; jlm80@psu.edu

**Keywords:** metal toxicity, metal resistance, acidophiles, metagenomics, metatranscriptomics, acidic systems, Cueva de la Mora, Iberian Pyrite Belt

## Abstract

Cueva de la Mora (CM) is an acidic, meromictic pit lake in the Iberian Pyrite Belt characterized by extremely high metal(loid) concentrations and strong gradients in oxygen, metal, and nutrient concentrations. We hypothesized that geochemical variations with depth would result in differences in community composition and in metal resistance strategies among active microbial populations. We also hypothesized that metal resistance gene (MRG) expression would correlate with toxicity levels for dissolved metal species in the lake. Water samples were collected in the upper oxic layer, chemocline, and deep anoxic layer of the lake for shotgun metagenomic and metatranscriptomic sequencing. Metagenomic analyses revealed dramatic differences in the composition of the microbial communities with depth, consistent with changing geochemistry. Based on relative abundance of taxa identified in each metagenome, Eukaryotes (predominantly *Coccomyxa*) dominated the upper layer, while Archaea (predominantly *Thermoplasmatales*) dominated the deep layer, and a combination of Bacteria and Eukaryotes were abundant at the chemocline. We compared metal resistance across communities using a curated list of protein-coding MRGs with KEGG Orthology identifiers (KOs) and found that there were broad differences in the metal resistance strategies (e.g., intracellular metal accumulation) expressed by Eukaryotes, Bacteria, and Archaea. Although normalized abundances of MRG and MRG expression were generally higher in the deep layer, expression of metal-specific genes was not strongly related to variations in specific metal concentrations, especially for Cu and As. We also compared MRG potential and expression in metagenome assembled genomes (MAGs) from the deep layer, where metal concentrations are highest. Consistent with previous work showing differences in metal resistance mechanisms even at the strain level, MRG expression patterns varied strongly among MAG populations from the same depth. Some MAG populations expressed very few MRG known to date, suggesting that novel metal resistance strategies remain to be discovered in uncultivated acidophiles.

## 1. Introduction

Water with elevated concentrations of dissolved metals is harmful to most life forms including animals, plants, and microorganisms. In general, heavy metal cations cause toxicity by promoting oxidative stress or by binding to protein sites not made for them, causing the proteins to malfunction [1]. Antioxidants such as glutathione protect cells from reactive oxygen species (ROS) and free radicals such as superoxide radicals [1]. Metals disrupt the balance between ROS production during aerobic energy metabolism and the subsequent defense provided by antioxidants. At high concentrations, ROS may cause structural damage to cells, proteins, nucleic acids, membranes, and lipids [1]. The mode of toxic action of the metalloid arsenic depends on its oxidation state. Arsenite, As(III), can bind to intracellular thiols such as glutathione and promote oxidative stress [2]. Arsenate, As(V), can disrupt the function of a variety of proteins (including those involved in ROS consumption) because of its structural similarity to phosphate [3]. Under acidic conditions, the speciation of arsenic is different from metal cations—i.e., As(III) is primarily an oxyanion and As(V) is primarily a neutral acid [4]. Because of the pH-dependent solubility of many minerals, systems with the highest concentrations of toxic metal(loid)s are usually acidic.

Acidophilic microorganisms employ a number of strategies to survive in the presence of high concentrations of metal(loid)s [5]. Metal resistance mechanisms include (i) transport (import/export), (ii) transformation, (iii) gene regulation, (iv) intracellular accumulation, and (v) extracellular sequestration [2]. Metal transport proteins typically function either to import an essential micronutrient (e.g., copper, iron, zinc) or to export a potentially toxic element (e.g., arsenic, cadmium, lead) [5]. Such metal import/export has to be tightly regulated to avoid intracellular metal overload. Regulation includes genes that repress or activate clusters of metal resistance genes (MRGs) to ensure metal homeostasis inside the cell [2].

A variety of metal resistance strategies involve import and/or export proteins. The expression of metal import genes can be constitutive or inducible. For example, the expression of *mntH*, a gene encoding for a Zn and Mn importer found in *Acidithiobacillus caldus*, *Acidimicrobium ferrooxidans*, and *Ferroplasma acidarmanus*, did not change after exposure to high zinc concentrations [6]. Limiting metal uptake can also be a mechanism for metal resistance. *Leptospirillum* species, for example, lack Fe(II) importers that could potentially allow Fe(II) to become toxic in environments with high Fe(II) concentrations [5]. Acidophiles typically have high numbers of metal export genes, many acquired by horizontal gene transfer [1]. One of the most studied bacterial acidophiles, *Acidithiobacillus ferrooxidans*, utilizes the Cop P-Type ATPases (CopAB) and the Cus CBA-transport system (CusABF) to export Cu out of the cell [7]. Orthologous genes for the Fe(II) exporter FieF were found in additional *Acidithiobacillus* and *Leptospirillum* strains [8]. The Co-Zn-Cd Czc RND export system has been identified in acidophiles such as *Leptospirillum* spp. [6]. The *ncrAC* genes encode membrane proteins that are part of a high-affinity nickel transport family were also found in a *Leptospirillum ferriphilum* strain [9]. The archaeon *Ferroplasma acidarmanus* upregulated the primary heavy metal exporter ZntA in the presence of excess of zinc [6], and also co-transcribed the *arsRB* operon involved in the export of As(III) in the presence of arsenic [10]. The As(III) efflux transporter, Acr3, was found in acidophilic green algae [11] such as *Coccomyxa* sp. Carn that transcribed genes homologous to *acr3* when exposed to arsenic [12]. As(III) is considered to be more toxic than As(V) so its export is critical [13].

Biochemical transformation of metals is another important metal resistance mechanism [5]. Many acidophiles reduce As(V) to As(III) in a reaction catalyzed by ArsC. The Fe(II)-oxidizing *Leptospirillum ferriphilum* as well as the sulfur-oxidizing *Acidithiobacillus caldus*, for example, both possess *arsC* containing operons and can survive in the presence of high arsenic concentrations (e.g., 5 mM As(III), 100 mM As(V)) [14,15]. The genomes of the acidophilic green algae *Chlamydomonas eustigma* and *Coccomyxa subellipsoidea* include the *arsC* gene that is highly expressed in *C. eustigma* under high arsenic concentration [11]. As(III) is then exported by the transporters ArsAB or Acr3. In contrast to eukaryotes and bacteria, archaea such as *Ferroplasma acidarmanus* that are resistant to As(V) lack ArsC, implying that they use a novel As(V) resistance/reduction mechanism [10]. As(III) can also be oxidized by the heterodimeric enzyme encoded by the *aio* gene. Aio has two subunits: AioA (molybdopterin) and AioB (Rieske protein). Such As(III) oxidase genes have been found in bacteria such as *Ralstonia* species [16] and in archaea such as *Sulfolobus tokodaii* [17]. Apart from detoxification, the *aio* gene cluster is also linked to electron transport [17].

Extracellular metal sequestration and intracellular metal accumulation also provide resistance for microorganisms dealing with high metal concentrations. Extracellular sequestration involves the immobilization of metal ions outside of the cell by interaction with metal chelators [18] or extracellular polymeric substances (EPS) [1]. EPS adsorb toxic metals directly or facilitate reactions involving the products of energy-related metabolisms (e.g., sulfate reduction produces H_2_S to form metal sulfides, or Fe(II) oxidation produces Fe(III) minerals that sorb or incorporate metals) [1]. Polysaccharides are essential constituents of EPS [19]. Among the polysaccharides important for biofilm formation and potentially extracellular metal sequestration are xanthan, sphingan, alginate, cellulose, and succinoglycan [20]. Succinoglycan imparted arsenic and mercury resistance in *Rhizobium* species [19] and cellulose produced by *Acetobacter* adsorbed Pb, Cu, Mn, Zn, and Co [21]. The precursor molecules for EPS polysaccharide synthesis are activated sugar acids assembled by glycosyltransferases and translocated across the cytoplasmic membrane by a Wzx-wzy dependent pathway or an ABC transporter dependent pathway [22]. Succinoglycan and xanthan are synthetized via the Wzx-wzy dependent pathway [20]. The ABC transporter dependent pathway is mainly involved in capsular polysaccharide biosynthesis [20]. Cellulose and alginate are synthesized via a third pathway involving a single synthase [20]. A fourth pathway involves extracellular production by a single sucrase protein (e.g., dextransucrase for the production of dextran) [20]. On the other hand, intracellular accumulation involves metal storage to decrease toxicity of metals that enter the cytoplasm [2]. *Acidithiobacillus* species make bacterioferritins (encoded by *bfr*) that bind to intracellular Fe [8]. Ferritins are able to sequester Fe by oxidizing Fe(II) and promoting the nucleation and growth of a ferric mineral [23].

Many genes encoding these diverse metal resistance mechanisms have yet to be identified, especially for acidophilic organisms [5]. BacMet [24] is a manually curated database of 753 bacterial genes that have been experimentally confirmed to confer resistance to metals and/or antibiotics. BacMet mainly includes genes involved in transport and transformation of metal(loid)s and their regulation. The most recent version of BacMet (2.0, March 2018) includes genes from a few model bacterial acidophiles such as *Leptospirillum ferriphilum*, *Acidithiobacillus ferrooxidans*, and *Acidiphilium multivorum*. Genes involved in synthesis of EPS are missing from BacMet as well as certain genes in the KEGG Orthology Database [25] annotated as important for Al, Mn, Fe, Ni, and As resistance (Table S1, KOs without BacMet IDs).

A better understanding of microbial metal resistance is needed to understand how microbial life adapts to and thrives in natural and industrial acidic environments. Such knowledge can contribute to the design of biotechnologies that use microbes for metal recovery and environmental remediation. For instance, in biooxidation tanks for gold recovery from arsenopyrite-containing ore, arsenic resistant acidophiles are needed to thrive under high arsenic concentrations reached during the process. Acidophiles in these systems must have or quickly acquire genes for arsenic resistance [5]. In bioleaching heaps, acidophiles must cope with high concentrations of metals such as Cu [5]. Addition of taxa known to contain arsenic or Cu resistance genes could facilitate horizontal gene transfer to the resident organisms of these systems to enhance their adaptation and performance [5]. Acidophiles can also be used in remediation of acid mine drainage (AMD), tailings ponds, or acidic pit lakes characterized by high metal concentrations of metals such as Fe, Cu, and Zn [5].

Cueva de la Mora (CM) is a permanently stratified (meromictic) acidic pit lake in the Iberian Pyrite Belt (IPB) in southwestern Spain. It has an upper oxic layer ca. 10 m deep, a sharp chemocline ca. 2 m thick, and a ca. 30 m thick anoxic layer. CM is one of the best studied pit lakes in the world, with over 15 scientific campaigns conducted between 2006 and 2018. Much research has been published in the last 10 years on different aspects of CM, including studies on its hydrology [26], physical limnology [27], aqueous geochemistry [28], colloid mineralogy [29], sedimentology [30], and microbiology [31,32]. CM is therefore an ideal model system to interrogate metal resistance mechanisms in an acidic pit lake using omics-enabled research. We compared microbial communities in all three layers of the lake, with a special focus on the deep anoxic layer because of its exceptionally high concentrations of toxic metal(loid)s.

The objective of this study was to develop a gene-resolved understanding of mechanisms that microorganisms in CM utilize to cope with metal toxicity imposed by the geochemical conditions characterizing each layer. To our knowledge, this is the first study using metagenomes and metatranscriptomes to relate microbial genes and gene expression with metal toxicity data under in situ conditions in an acidic pit lake. We identified the most toxic metal(loids) based on existing toxicology data and then quantified the normalized abundance of genes and transcripts involved in metal resistance from microbial communities (upper oxic layer, chemocline, deep anoxic layer). In the especially metal-rich deep layer, we recovered metagenome-assembled genomes (MAGs) to test whether different species populations employ different metal resistance mechanisms in the same environment.

## 2. Materials and Methods

### 2.1. Geologic Setting

The Iberian Pyrite Belt (IPB) is a world-class metallogenic province located in the southwestern corner of the Iberian Peninsula, extending from north of Seville, Spain to south of Lisbon, Portugal. The IPB has been mined since ca. 3000 B.C. [33] and most intensively from ca. 1890–1990 [34]. Modern mechanized open cast mining operations during the 1960–1990s left over 20 pits that have since been abandoned and flooded [35]. These acidic pit lakes share a common geologic framework defined by the ubiquitous presence of pyrite, abundant aluminosilicates, and usually a scarcity of carbonate minerals.

Cueva de la Mora (CM) contains dramatic vertical gradients of redox conditions and water composition (Table 1; Figure S1). After the cessation of mining in 1971, the pit flooded and developed permanent stratification. At present, CM has an estimated volume of 282,300 m^3^, a surface area of 17,800 m^2^, and a maximum depth of 40 m. CM is stable in terms of hydrogeochemical conditions. The anoxic layer is relatively isothermal (18 °C) compared to the upper layer (8–28 °C depending on season). The anoxic deep water also shows higher pH (pH 3.8–4.3 vs. 2.1–2.7), lower Eh (300 mV vs. 800 mV), and higher concentrations of dissolved solids (e.g., specific conductance (SC) of 10–12 vs. 2–4 mS/cm) compared to the upper oxic layer. Although the vertical trends in these parameters show slight seasonal variations, the general features are constant throughout the year.

### 2.2. Sample Collection, Physico-Chemical Profiling/Field Data Acquisition and DNA/RNA Extraction

In May 2018, field data and physicochemical profiles of temperature, pH, Eh, and specific conductance were acquired with a MS5 datasonde from Hach (Loveland, CO, USA) previously calibrated according to the manufacturer’s instructions. The goal of this monitoring was to ensure the physical and chemical stability of the pit lake with respect to previous campaigns (Figure S1). Water samples were then collected along a depth profile above the deepest part of CM, specifically at 3, 11, and 35 m representing the upper oxic layer (identified as CM03), chemocline (CM11) and deep anoxic layer (CM35), respectively. Three samples (each one representing a biological replicate) were collected with a 5 L Van Dorn limnological ‘horizontal’ sampling bottle (KC Denmark A/S, Silkeborg, Denmark) per depth. For each sample, 1 L of water was prefiltered through a 2 µm pore size glass fiber membrane and subsequently filtered in a 0.22 µm polyethersulfone (PES) sterivex filter immediately after collection for RNA extraction. Up to 3 L of water were filtered for DNA extraction. Filters were cryo-shipped to the US and preserved at −80 °C until DNA and RNA extraction. Filters were excised under aseptic conditions and added to a lysing matrix tube that underwent DNA extraction using the Qiagen DNAeasy Powerwater Kit and RNA extraction using the Qiagen RNeasy PowerMicrobiome Kit (Qiagen, Venlo, The Netherlands). DNA extracts were quantified using the Qubit^®^ 2.0 Fluorometer (Invitrogen, Carlsbad, CA, USA). RNA extracts were quantified using both the Qubit RNA high sensitivity and DNA high sensitivity Fluorometer (Invitrogen, Carlsbad, CA, USA) as well as a Bioanalyzer 2100 RNA 6000 Pico Assay (Agilent, Santa Clara, CA, USA).

### 2.3. Metagenomics and Metatranscriptomics Sequencing

Two biological replicates per depth with the most DNA and RNA concentrations and quality were selected for library preparation and sequencing. Metagenome library preparation was performed using Illumina’s NexteraXT library preparation kit (Illumina, San Diego, CA, USA) with 1 ng of total genomic DNA as starting material, tagmented with Illumina adapters and unique 8 bp dual indices, and 12 PCR cycles. Double stranded cDNA synthesis and metatranscriptome library preparation was performed using the Tecan RNA Trio library preparation kit (Tecan, Mannedorf, Switzerland) with 10 µL of RNA as starting material and two rounds of PCR, the first one with 6 PCR cycles and the second one with 8 PCR cycles. The metatranscriptome library preparation kit is designed for low-input RNA and did not include a ribosomal RNA depletion step. Therefore, total RNA was prepared for metatranscriptomes and in silico rRNA removal was conducted prior to downstream analysis (see Section 2.4). Libraries were normalized and multiplexed using a fragment size between 250–400 bp. The metagenome and metatranscriptome libraries were then purified on a 2% agarose gel and size selected using the QIAquick gel extraction kit (Qiagen, Venlo, The Netherlands). Sequencing was conducted on an Illumina Hiseq 4000 platform (Illumina, San Diego, CA, USA) using 150 bp paired end chemistry. Library preparation and sequencing was conducted by an external core-genomics facility. Two metagenomic and two metatranscriptomic datasets were obtained per sampling depth. The upper layer metagenomes had a total of ~134 million raw reads, and its metatranscriptomes had a total of ~52 million raw reads. The chemocline metagenomes had a total of ~92 million raw reads, and its metatranscriptomes had a total of ~45 million raw reads. The deep layer metagenomes had a total of ~130 million raw reads, and its metatranscriptomes had a total of ~106 million raw reads.

### 2.4. Whole Community Metagenome and Metatranscriptome Processing

Raw reads from metagenome and metatranscriptome datasets were quality-filtered with Trimmomatic v0.36 with a minimum Phred quality score of 33 [36]. Nonpareil 3 [37] was used to estimate the metagenomic coverage; all metagenomes had more than 80% coverage. Quality-filtered metagenomic reads were individually assembled and co-assembled with Megahit v1.1.2 with default parameters [38]. EMIRGE [39] was used to recover and reconstruct 16S and 18S rRNA sequences from the metagenomes. SINA v.1.2.11 was used for taxonomic classification of the reconstructed SSU sequences to evaluate microbial community compositions [40]. Detected 16S rRNA sequences from chloroplasts were removed prior to calculation of relative abundance. Relative abundance of the identified taxa was calculated based on the number of reads from the metagenomes mapped to the EMIRGE-reconstructed SSU sequences. Due to the presence of eukaryotic organisms in CM03 and CM11, eukaryotic contigs from co-assembled metagenomes were selected with EukRep [41] and genes were predicted with MetaEuk [42] using the NCBI-nr database (downloaded May 2020) [43]. Prodigal v2.6.3 [44] was used to predict genes (open reading frames) in bacterial and archaeal contigs from co-assembled metagenomes. Predicted genes were deduplicated with dedupe.sh [45]. Reads from the individual metagenomes were mapped to the predicted genes from the respective co-assembled metagenomes with BBmap (min_id = 0.95, slow mode) [46], and TPM (transcripts per million) per predicted gene was calculated as a proxy for gene abundance (TPM = number of reads from metagenome mapped to one gene (×10^6^) divided by its length/sum of number of reads from metagenome mapped to all genes divided by respective lengths). Taxonomic annotation was conducted with DIAMOND v0.9.32.133 [47] against the NCBI-nr database [43] using 50% identity over at least 80% length as the annotation threshold. The top hits per predicted gene were visualized with Megan v6.18.10 [48]. Predicted genes were also annotated with GhostKOALA [49] and KOFAMscan v1.3.0 [50] for functional annotation. Around 50% of predicted genes in the three co-assembled metagenomes were assigned to a KEGG Orthology identifier (KO) with an E-value < 10^−4^. In silico removal of ribosomal RNAs was conducted with sortmeRNA v2.1 [51] in the quality-filtered metatranscriptomic reads. The quality-filtered remaining reads (mostly mRNAs) were mapped to the co-assembled metagenomic contigs with BBMap [46] (min_id = 0.95 and slow mode). Around 50% or more of mRNA reads were mapped to the respective metagenome contigs, except for one metatranscriptome from CM35_2 (<1%) that was not used for downstream analysis. The mRNAs from each metatranscriptome were also mapped to the predicted genes from the co-assembled metagenomes, and TPM values were calculated as proxies for transcript abundance. The gene and transcript profiles were converted to functional profiles by summing the normalized abundances of the predicted genes part of the same functional group (KEGG Orthology-KO). Expression was calculated as a ratio between KO-transcript abundance and KO-gene abundance (RNA_TPM/DNA_TPM per KO). General statistics of metagenomes and metatranscriptomes from each layer are detailed in the Supplementary Information (Table S2).

### 2.5. Metagenome-Assembled Genome Processing

Different approaches were applied to obtain metagenome-assembled genomes (MAGs) from the deep layer (CM35). MaxBin v2.2.4 (with default parameters except for -min_contig_length = 1500, -prob_threshold 0.95, and -markerset 40 for archaeal dominated samples) [52] and MetaBat v2.12.1 (with default parameters) [53] were used over the individual assembled metagenomes, the co-assembled metagenomes, and a subset of assembled reads that represented 100% coverage of the metagenomes. DASTool [54] and dRep v2.3.2 [55] were used with default options to select the best quality and de-replicated MAGs. FastAni v1.3 [56] was utilized to select those MAGs with an average nucleotide identity (ANI) of <96.5%. Relative abundances (Rel. Abu.) were calculated based on total DNA-reads mapped to each MAG. Prodigal v2.6.3 [44] was used to predict genes (open reading frames) from each MAG. The predicted genes were annotated with KOFAMScan v1.3.0 [50]. Taxonomic assignation of the MAGs was conducted with GTDBT-k 1.1 [57]. The quality-filtered DNA reads (metagenome) and mRNA reads (metatranscriptome), both coming from the same sample (CM35_1), were mapped to the predicted genes of each MAG with BBmap (min_id = 0.95, slow mode) [46], and TPM per predicted gene was calculated to obtain normalized gene abundance and transcript abundance per MAG (number of reads from metagenome mapped to gene (×10^6^) divided by length/sum of number of reads from metagenome mapped to all genes divided by length per MAG). The gene and transcript profiles were converted to functional profiles by summing the normalized abundance of the annotated genes part of the same functional group (KO). Expression was calculated as KO-transcript abundance divided by KO-gene abundance.

### 2.6. Metal Resistance Gene Database

We built a list of marker KOs for metal resistance mechanisms. We first downloaded the 753 experimentally confirmed resistance genes from BacMet database v2.0 [24], updated March 2018, from which 485 were MRGs. To designate KOs for the BacMet genes, we annotated them with KOFAMScan [50] and selected those that had scores higher than the threshold (best hits) and obtained a list of BacMet genes with assigned KOs. Since the BacMet database included genes with different BacMet IDs corresponding to the same function but different taxonomic assignation, we aggregated our marker gene list based on their KOs, and manually typed all the compounds/metals that the BacMet database described for all BacMet genes with the same KO. We then down-selected the genes involved only in metal resistance whose BacMet gene description matched the KO description and obtained a list of 134 MRGs with both KO and BacMet identifiers.

KOs with functions involved in the assembly and transport of EPS as well as glycosyltransferases involved in the synthesis of certain EPS (succinoglycan, xanthan, colanic acid, alginate, cellulose, and dextran) were considered as proxies for extracellular metal sequestration. Among the KOs involved in the assembly and transport of EPS, KOs that are part of the Wzx-wzy dependent and independent (ABC-transport) pathways were included in our marker list. Most of these genes have been reported in Gram-negative bacteria and are reviewed in [20,58,59]. Four KO groups were considered for the Wzx-wzy dependent pathway: polymerases (wzy-like), flippases (wzx-like), polysaccharide co-polymerases (PCPs), and outer membrane transport proteins (OPXs). Among the KOs involved in the ABC-transport of EPS, two ABC-transporters (*kpsTM*) and a PCP (*kspE*) were included in our marker list. A KO for the extracellular synthesis of dextran (*dps*) was also included in our marker list.

Other genes reported in [2,60,61,62,63,64] that were not part of the BacMet database and had an assigned KO were also included in the marker KOs list. Our final list of marker KOs for metal resistance included a total of 222 MRGs (Table S1). Using a script in R (https://github.com/DianaKarina/MRG_IPBlakes), we selected the genes annotated with KOFAMscan with an E-value < 10^−4^ [50] from metagenomes and MAGs in accordance with the KOs matching our marker KOs list for downstream analysis.

### 2.7. Data Availability

Raw data for metagenomes and metatranscriptomes are available in the SRA database as bioproject PRJNA646106. This bioproject also includes the MAGs in FASTA files.

## 3. Results and Discussion

### 3.1. Toxic Potency Factors

While there are many toxic elements in Cueva de la Mora (CM), and at different concentrations in the different layers, it is challenging to predict which elements may exert the greatest stress on the microbes in each layer. One approach is to review and compare studies where a single indicator species (e.g., the bioluminescent bacterium *Aliivibrio fischeri* or the planktonic crustacean *Daphnia magna*) is incubated with each of these toxic elements and then rank their potency based on their half maxima effective concentration (EC_50_ values). There are several challenges with adopting this approach. First, we found that the ranked potencies differ in studies using the same indicator species, highlighting how results could be affected by background electrolytes, temperature, pH, or other variables among the existing studies. We also found no single study that tested all of the elements we presumed were the most toxic in CM (Al, As, Co, Cu, Fe, Mn, Ni, and Zn).

An alternative approach to rank metal toxicity is to base it on water quality criteria. For example, USEPA National Ambient Water Quality Criteria (NAWQC) include numerical limits for in-stream dissolved concentrations of ~20 elements, including all of the presumed most toxic elements but Mn. These numerical limits were set based on protecting human health and freshwater aquatic organisms. Using these numerical limits, we defined a dimensionless toxic potency factor (TPF-1) as

TPF-1 = measured dissolved concentration/NAWQC − CCCF standard(1)

where the measured dissolved concentration (µg/L) did not account for speciation and the NAWQC-CCCF standard (µg/L) was based on a constant contact concentration factor (CCCF) (i.e., continuous exposure). For several metals, USEPA uses additional adjustments to the NAWQC-CCCF standard values that depend on the hardness of the water. None of the hardness-based adjustments were used in our TPF calculations.

Metal toxicity has been shown to be better correlated to the activity of the free metal cation as compared to the dissolved concentration of the metal [65]. Therefore, a second toxic potency factor (TPF-2) was calculated as

TPF-2 = free cation activity/NAWQC − CCCF standard(2)

where the free cation activity was calculated from speciation modeling (using PHREEQC) for each lake layer. Based on these operationally-defined estimates of toxic potency, we found that different elements were predicted to be most toxic in the different layers (Table 2). Specifically, we found that Cu and Al were expected to be most toxic in the upper oxic layer (CM03) whether calculated based on dissolved concentration or free cation activity. For the chemocline, toxicity rankings depended on whether they were based on dissolved concentrations (Al more toxic than Fe(II)) or free cation activities (Fe(II) more toxic than Al). Fe(II) was predicted to be most toxic in the deep layer because of its exceptionally high concentration. Zn and Mn were predicted to be toxic in all three layers. Toxic potencies for As were only predicted to be substantial in the deep layer. Speciation modeling predicted that As would be ~100% As(V) in the upper layer and chemocline, and ~100% As(III) in the deep layer. We hypothesized that these differing toxic potencies would be reflected by differing expression of metal-specific gene-based responses from the microbial communities in each lake layer.

### 3.2. Microbial Diversity

**Figure 1:**
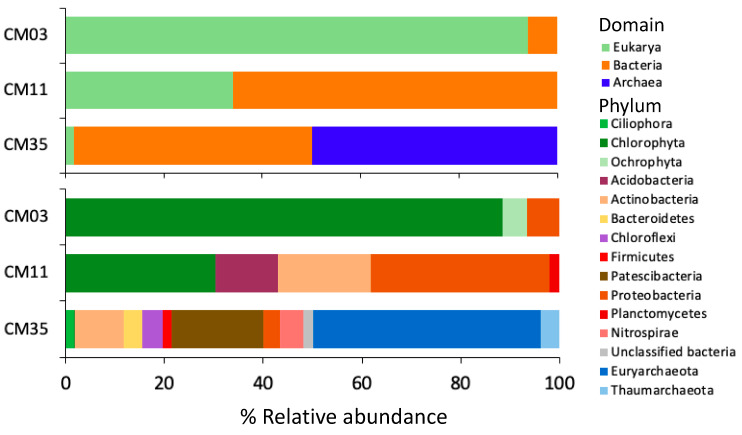
Taxonomy of the three layers of Cueva de la Mora (CM) at the domain (upper panel) and phylum (lower panel) levels. 16S and 18S rRNA genes were recovered from the metagenomes using EMIRGE [39] and SINA v.1.2.11 [40] for taxonomic classification.

The reconstruction of 16S and 18S rRNA sequences from the metagenomes using EMIRGE [39] revealed the dominance of eukaryotic microorganisms in the upper layer, bacteria in the chemocline, and archaea in the deep layer (Figure 1, Table S3). Eukaryotes were also abundant in the chemocline. Bacteria were found at all depths, whereas archaea were only retrieved from the deep layer. Single-celled eukaryotes from the phylum *Chlorophyta* were the most abundant eukaryotes and had relative abundances of 88% and 30% in the upper layer (CM03) and chemocline (CM11), respectively. Sequences assigned to *Chlorophyta* had an identity of 98% to species in the phototrophic genus *Coccomyxa* (Table S3). The acidophile *Coccomyxa onubensis* was previously isolated from water collected in the upper layer of CM [32] and has also been found in other acidic waters (e.g., the Tinto River [66]). The most abundant bacteria in the upper layer were associated to an unclassified bacterium from the genus *Acidiphilium*, phylum *Proteobacteria*, class *Alphaproteobacteria*, with a relative abundance of 7%. Organisms associated with this genus have been previously isolated from the chemocline of CM, but have not been reported in the upper layer [32]. *Acidiphilium* species are known to be facultative anaerobes and heterotrophs that can reduce Fe(III) under anaerobic conditions [32]. Other acidophilic heterotrophs present in the upper layer include *Acidobacteriaceae* and *Acidisphaera rubrifaciens* [32] but their 16S rRNA sequences were not retrieved by EMIRGE probably due to their low abundances.

In addition to large populations of *Coccomyxa* (30%), the chemocline hosts major populations of bacteria, consistent with a dynamic, metal-rich, redox-gradient interval. Fe(II) oxidation and Fe(III) reduction, as well as sulfide oxidation and sulfate reduction have previously been documented in the CM chemocline [31]. The most abundant bacteria in the chemocline sample were related to uncultured *Actinobacteria* in the class *Acidimicrobia* (19%), to non-spore forming heterotrophic sulfate reducers in the *Deltaproteobacterial* genus *Desulfomonile* (18%), to uncultivated SVA0485 *Deltaproteobacteria* (18%), and to heterotrophic ferric iron reducers in the *Acidobacteria* genus *Acidicapsa* (13%) (Figure 1 and Table S3). Our results are consistent with previous work at CM that reported major populations of *Desulfomonile* in the CM chemocline [32].

The anoxic deep layer hosted abundant archaea dominated by *Euryarchaeota* and bacteria affiliated with phyla including *Actinobacteria*, *Bacteroidetes*, *Chloroflexi*, *Firmicutes*, *Nitrospirae*, *Patescibacteria*, and *Proteobacteria*, and was overall more diverse than the upper layer and chemocline. Uncultivated *Thermoplasmatales-group “I-plasma”* populations were the most abundant archaea and the most abundant populations in the deep layer overall (35%, Table S3). Uncultured *Gaiellales* sp. (*Actinobacteria*, class *Thermoleophilia*), *Candidatus Roizmanbacteria* (*Patescibacteria*, class *Microgenomatia)*, and *Candidatus Adlerbacteria* (*Patescibacteria*, class *Parcubacteria*) were among the most abundant bacterial populations with 8%, 8%, and 7% relative abundance, respectively. In contrast to our results, only archaeal DNA was previously amplified from this layer [32]. With such distinctly different microbial communities in each lake layer as well as extreme gradients in geochemistry, we hypothesized that metal-resistance mechanisms in the CM microbial communities would display correspondingly strong differences.

### 3.3. Metal Resistance Mechanisms

**Figure 2:**
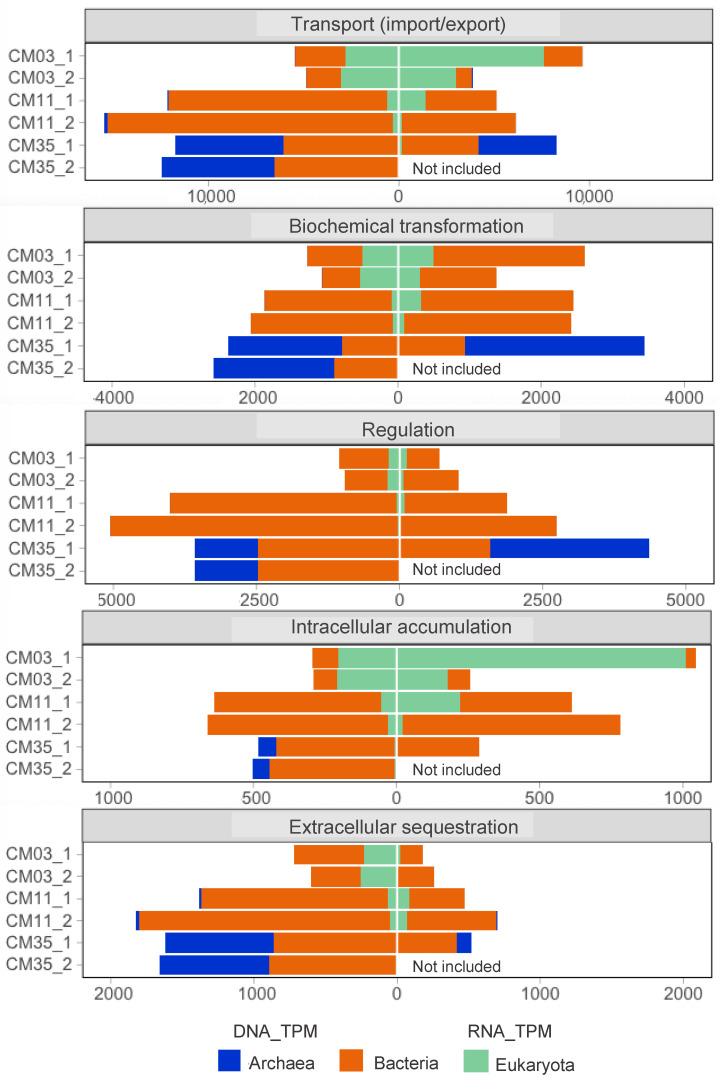
Gene profiles (left sides) and transcript profiles (right sides) of metal resistance response mechanisms detected in the three layers of Cueva de la Mora (CM) and mapped back to the domain level. Genes per metagenome were functionally annotated with KOFAMscan [50] and taxonomically annotated with Diamond + MEGAN [47,48] and GhostKOALA [49]. _1 and _2 refer to replicates (DNA or mRNA reads from each replicate mapped to co-assembled metagenomes). Gene and transcript abundances calculated as TPM values. List of KOs for each metal resistance mechanism provided in Table S4. mRNA reads from CM35_2 are not included due to low mapping rates to the respective co-assembled metagenome.

Metagenomics and metatranscriptomics results are presented in terms of five metal resistance mechanisms mapped back to the domain level in each layer of CM (Figure 2). Metal resistance mechanisms included transport (import/export), biochemical transformations, regulation, intracellular accumulation, and extracellular sequestration. As noted above in the Materials and Methods section, we used 222 MRGs (Table S1) and assigned each marker KO to one of these five metal resistance mechanisms. From the annotated genes with KOFAMscan (E-value < 10^−4^) in each metagenome, ~3% were annotated as MRGs. Normalized gene and transcript abundance are presented in terms of TPM values (DNA_TPM and RNA_TPM, respectively). In general, we observed that the gene-encoded functional potential for the five analyzed mechanisms of metal resistance increased with depth corresponding to greater metal concentrations at depth (Figure 2 and Table 1). Of the five metal resistance mechanisms, transcripts for metal transport were the most abundant, particularly in the upper and deeper layers (Figure 2). The most abundant transcripts from eukaryotes were annotated in functions related to metal transport (most abundant transcripts corresponded to *acr3* and *pho84*) and intracellular accumulation (*fth1*). In contrast, the most abundant transcripts from archaea were mainly related to biochemical transformations (*aioB*/*aoxA* and *rus*) and regulation (*mntR* and *troR*). Transcripts from bacteria were associated with all five mechanisms.

### 3.4. Element-Specific Response Mechanisms

Many marker KOs in our compilation of MRGs were noted to interact with only one element. Other marker KOs noted to interact with two or more elements are referred to herein as non-specific MRGs. To focus our analysis, we selected seven metal(loid)s with the highest predicted toxicities (Table 2) and 48 KOs related to genes conferring resistance to such metals (Table 3). From the 222 MRGs compiled in our database, we focused on these 48 MRGs to evaluate how their gene-encoded potential and expression corresponded to our predictions of toxic potency factors (Table 2).

**Figure 3:**
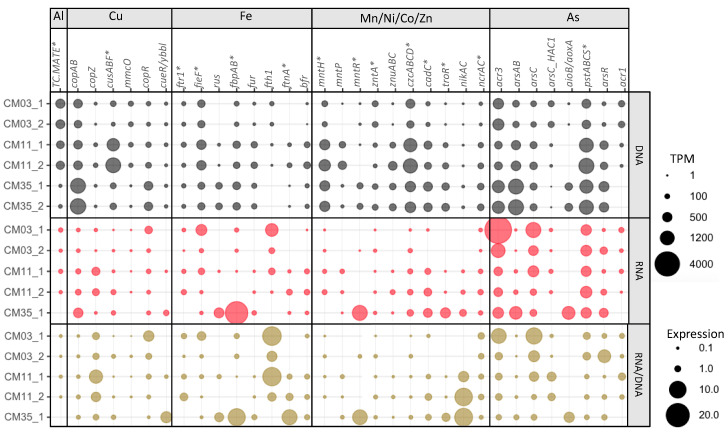
Normalized gene abundance (upper rows), normalized transcript abundance (middle row), and expression (lower rows) of metal-specific metal resistance genes (except with *) detected in the three layers of Cueva de la Mora (CM). Normalized gene and transcript abundances are calculated as TPM values. Expression is calculated as transcript TPM/gene TPM. 1 and 2 refer to replicates (DNA or mRNA reads from each replicate mapped to co-assembled metagenomes). Gene functions and their respective KOs are listed in Table 3. mRNA reads from CM35_2 are not included due to low mapping rates to the co-assembled metagenome.

Expression patterns for metal-specific MRGs did not typically correlate with dissolved metal concentrations (Figure 3). Increased MRG expression with increased metal concentrations may have been uncommon (Figure S2) because TPM values and expression ratios for the whole communities reflect both taxonomic and geochemical differences between the layers. However, the expression patterns for some genes involved in resistance to Al (*TC.MATE*, also described as *SLC47A*, *norM*, *mdtK*, *dinF*), Mn (*mntP*), Zn (*zntA*), and As (*arsAB*) were positively correlated to the corresponding metal(loid) concentrations (Figure S2). In contrast, the expression patterns for some genes involved in resistance to Cu (*copAB*), Fe (*fieF*), Ni (*ncrA*), and As (*acr3* and *arsC*) were negatively correlated to metal(loid) concentrations. Positive or negative refers only to the slope of the regression and not statistical significance. In the case of Al, higher concentrations were found in the upper layers coincident with higher expression of *TC.MATE* (Multidrug and toxic compound extrusion (MATE) family genes, potentially involved in Al tolerance [67]) which were mapped mainly to eukaryotes. In the case of As, the upper layer showed higher expression of *acr3* (an As(III) export gene) than the other layers (Figure 3) despite that speciation modeling predicted little As (III) in this layer (Table 2). Consistent with speciation predictions, the As(V) reduction genes *arsC* were expressed more in the upper layer and mapped mainly to bacteria. Expression patterns in the upper layer are consistent with intracellular As(V) reduction (using *arsC*) followed by As(III) export (using *acr3*). In contrast, the *arsAB* genes (also involved in As(III) export) were expressed more in the deep layer and mapped mainly to bacteria and archaea, consistent with high concentrations of As(III) in the deep layer.

#### 3.4.1. Aluminum

Aluminum is one of the metals with high dissolved concentrations and toxic potency factors in the upper layer and chemocline (Table 2). A significant challenge for assessing microbial responses to Al is a lack of marker genes. In the BacMet database [24], the genes BAC0489 (*ALU1-P*) and BAC0490 (*G2alt*), annotated as a 7-cyano-7-deazaguanine synthase (K06920) by KOFAMscan, are Al-specific. Such genes were detected in the chemocline of CM at low frequency and without expression. Therefore, genes from the MATE family (*TC.MATE*, K03327), a family of multidrug and toxic compound extrusion transporters known to be involved in tolerance to Al and pharmaceuticals [67], were added to our KO marker list. These transporters are present in the three domains of life. The bacterial MATE transporters interact with cationic compounds, including antibiotics, and some plant MATE transporters confer Al tolerance to plants in acidic soils [67]. Consistent with geochemical measurements showing that Al concentrations are higher in the upper layers, the gene-encoded potential and expression of *TC.MATE* decreased with depth (Figure 3). It is important to note that we are likely missing key Al-specific MRGs because such genes have not yet been widely described.

#### 3.4.2. Copper

Metagenomes from all layers contained the genes for all of the Cu-specific genes, however, diversity and expression of Cu-specific genes was greatest in the chemocline. Toxic potency factors were far higher in the upper layer as compared to the chemocline (Table 2). Our results showed increased normalized abundance of genes and transcripts involved in Cu export (*copAB* and *cusABF*) but their expression was similar in all three layers (Figure 3). According to the BacMet database, some of the genes annotated as *copA* (K17686) also confer resistance to sodium acetate and silver (Ag) and the genes *cusABCF* are also involved in Ag(I) export. The Cu chaperone gene (*copZ*), which acts in association with Cu-transporting ATPases (*copA*), was expressed along depth with higher expression in the chemocline. The expression of such genes (P-type transporters and Cu chaperones) in the chemocline and deep layer might also be involved in transfer of other divalent cations such as Cd^2+^, Co^2+^ and Zn^2+^ [70]. Genes encoding a multicopper oxidase (*mmcO*) were retrieved from metagenomes from all three layers, but *mmcO* was not expressed in the deep layer potentially because of the reducing conditions. The Cu resistance response regulator (*copR*) had higher expression in the upper layer, in contrast with the Cu efflux regulator (*cueR*/*ybbl*) which was mainly expressed in the deep layer. Another mechanism of Cu resistance employs degradation of polyphosphates followed by export of Cu-phosphate complexes [71]. Genes involved in this mechanism (*ppx*, *pitA*, and *pho84*) were included in our analysis. The *pho84* gene was more expressed in the upper layer, but the other genes were similarly expressed in the three layers (Table S4), not responding exclusively to the high toxicity of Cu in the upper layers.

#### 3.4.3. Iron

As with Cu-specific genes, the chemocline community expressed all of the Fe-specific resistance genes (Table 3, Figure 3). Genes related to the Fe(II) export can also export Zn/Co/Cd (Table 3). These genes might confer resistance to divalent cations in the upper layer, but may be more involved in Fe(II) export in the chemocline and deep layer given the predominance of Fe(II) and its predicted toxicity in these layers (Table 2). Previous studies have reported that acidophiles contain Fe homeostatic systems similar to neutrophiles and may even lack mechanisms to export Fe(II) [5]. The *rus* gene, well-known for its role in aerobic Fe(II) oxidation [72], is expressed in the deep layer. Although the concentration of Fe(II) increased with depth, the anoxic conditions of the deep layer may not promote Fe(II) oxidation. Fe(II) may induce the active form of rusticyanin [73] but its role could be more related to either Fe transport or oxidative stress in the deep layer [74]. The genes *fbpAB* code for an Fe(III) transport system, specifically Fe(III) import, and were also highly expressed in the deep layer. Expression of the *fth1* gene, which encodes ferritin in eukaryotes, suggests that the dominant eukaryotes in the mixolimnion may store Fe intracellularly. Homologues to *fth1* have been found in *Coccomyxa subellipsoidea* [75]. Two Fe(II) related resistance genes, *ftnA* and *bfr*, were expressed in the chemocline and deep layer. These enzymes reduce the concentration of free Fe(II) in the cytoplasm by oxidizing it and storing it as Fe(III) [76]. We expected to see higher expression of Fe(II) export genes in the deep layer, but instead observed a higher expression of Fe(III) uptake genes and Fe storage. This result implies that this microbial community might employ other genes for Fe(II) resistance or that Fe(II) was not as highly toxic as our toxicity potency factors suggested. Tolerances to Fe(II) are usually much higher than to Fe(III) in acidophiles [77,78].

#### 3.4.4. Manganese, Nickel, Cobalt, and Zinc

Genetic potential and expression of genes conferring resistance to Mn, Ni, Co, and Zn were consistent with increasing concentrations at depth (Figure 3). Microbial communities in all the layers can export Mn (*mntP*). Expression of the Mn transport regulator *mntR* in the deep layer is consistent with higher Mn concentrations at this depth. The *mntR* genes are transcriptional repressors that regulate the metal uptake expression by sensing the concentration of Mn(II) [69]. In *Escherichia coli*, *mntR* represses *mntH* (Mn importer) and positively regulates *mntP* (Mn exporter) [79]. Similarly, expression of *troR*, another Mn-dependent regulator but also involved in Fe and Zn resistance, increased at depth. Zn was expected to exert high toxicity in the deep layer and genes and transcripts involved in Zn extrusion (*czcABCD*) and regulation (*cadC* and *troR*) were more abundant at depth. Finally, expression of the Ni/Co transporters *nrcAC* were similar along depth consistent with the toxic potency of Ni not differing much between layers. Genes involved in Ni transport (*nikAC*) were, however, more expressed in the chemocline and deep layer.

#### 3.4.5. Arsenic

Genetic potential and expression for the majority of the As-specific genes were found in all three layers (Figure 3). Even though As concentrations are over two orders of magnitude lower in the upper layer compared to the deep layer, genes involved in As export and biochemical transformation were highly expressed in the upper layer. Detection of genes involved in As(III) export (*acr3*), As(V) import (*pstABCS*) and As(V) reduction (*arsC* and *arsC_HAC1*) suggest that certain microorganisms could potentially uptake As(V), reduce it to As(III) and export the latter for detoxification purposes. The *aioB/aoxA* genes encode for the small subunit of an As(III) oxidase protein. The As(III) oxidase requires an extra subunit, encoded by the *aioA*/*aoxB* gene, for its catalytic activity that was not found in our metagenomes. The AoxA protein contains a ‘Rieske’ [2Fe-2S] center to which electrons are transferred from AoxB then transferred to the respiratory chain towards a terminal electron acceptor that could be oxygen, nitrate, or chlorate in bacteria and oxidize As(III) [2]. Although not involved in detoxification purposes, the role of *aioB*/*aoxA* genes in electron transfer for respiratory purposes could be relevant for the microbial community of the deep layer.

#### 3.4.6. Other Metal Response Mechanisms

There are other mechanisms in acidophiles that can contribute to their metal resistance. Acidophiles can maintain an internal positive membrane potential to prevent protons and metals from entering the cell [5]. Acidophilic bacteria, for example, display intrinsic resistance to the influx of metals by accumulating anions (e.g., chloride) especially at low pH [80]. Metal speciation and sorption at low pH can also help acidophiles survive. High sulfate concentrations typical in acidic systems promote metal complexation and decrease activity of free metal cations (e.g., speciation results in Table 2). Finally, competition between protons and free metals for binding sites on cell surfaces could also decrease metal toxicity [81].

### 3.5. Extracellular Metal Sequestration

Previous studies using high-resolution scanning/transmission electron microscopy of samples collected from various depths in CM showed cells encrusted with mineral precipitates [82,83]. Extracellular polymeric substances (EPS) containing polysaccharides can sorb metals, providing another mechanism for microbial metal resistance [82]. We considered KOs related to the biosynthesis, assembly, and transport of extracellular and capsular polysaccharides as a proxy for extracellular metal sequestration that may promote mineral precipitation (Table 4). Based on these marker genes, both gene and transcript abundance associated with extracellular metal sequestration increased with depth (Figure 2). Bacteria in the chemocline and deep layer, and archaea in the deep layer all expressed genes involved in this mechanism of metal resistance.

**Figure 4:**
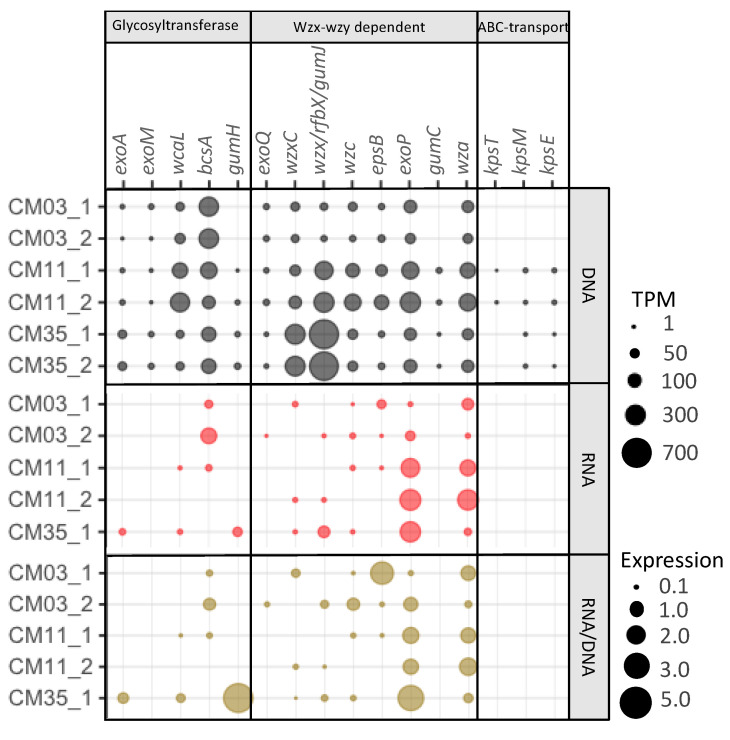
Normalized gene abundance (upper rows), normalized transcript abundance (middle row), and expression (lower rows) of extracellular sequestration genes detected in the co-assembled metagenomes from the three layers of Cueva de la Mora (CM). Normalized gene and transcript abundances are calculated as TPM values. Expression is calculated as transcript TPM/gene TPM. All of these genes are involved in biosynthesis of polysaccharides (glycosyltransferases), and polysaccharide assembly and transport (Wzx-wzy dependent pathway and ABC transport). 1 and 2 refers to replicates (DNA or mRNA reads from each replicate mapped to co-assembled metagenomes). Gene functions and their respective KOs are listed in Table 4. mRNA reads from CM35_2 are not included due to low mapping rates to the co-assembled metagenome.

Among the KO markers for extracellular metal sequestration, those coding for glycosyltransferases and proteins involved in assembly and transport of polysaccharides by the Wzx-wzy dependent pathway were expressed (Figure 4). Genes coding for the glycosyltransferases WcaL (colanic acid/amylovoran synthesis) and BscA (cellulose synthesis) were abundant along depth. Transcripts for *bscA* were relatively high in the upper layer and chemocline. Other genes coding for glycosyltransferases involved in the synthesis of succinoglycan (*exoA*) and xanthan (*gumH*), two EPS polysaccharides reported to be important in metal resistance [19], were found in all metagenomes but transcripts were only identified in the deep layer. Among the KOs involved in the Wzx-wzy dependent pathway for assembly and transport, *exoQ* was the only polymerase gene identified in the metagenomes. Genes coding for flippases (*wxcC*, *gumJ*) were more abundant in the deep layer metagenomes, where transcripts were also found. The genes coding for polysaccharide co-polymerases (PCP), *epsB* and *exoP*, were found in all metagenomes, but more abundant transcripts for *exoP* (involved in succynoglycan biosynthesis) were found in the chemocline and deep layer. Among the outer membrane transporters (OPX), *wza* was the only one identified in all the metagenomes and metatranscriptomes, with higher expression in the chemocline. Finally, the KOs involved in the ABC-transport pathway for assembly and transport of EPS were not abundant in the metagenomes and not expressed at all in the metatranscriptomes. Based on these results, the microbial community in the upper layer has the potential and activity for synthesis of cellulose. In the chemocline, the microorganisms have potential and activity for synthesis of colanic acid and cellulose. The deep layer microorganisms have potential and activity for synthesis of succinoglycan, colanic acid, and xanthan. All three communities have the Wzx-wzy dependent pathway for EPS assembly and transport. In previous studies, however, heterotrophic acidophiles isolated from the upper layers of CM did not promote extracellular sequestration of Fe or Al under laboratory conditions, except for *Acidibacter ferrireducens* that showed extracellular precipitation when grown in the presence of Fe [85]. Therefore, uncultivated microorganisms in CM might be involved in extracellular metal precipitation.

### 3.6. Metal Resistance Mechanisms in Deep Layer Populations

We used metagenome-assembled genomes (MAGs) to explore whether different populations use different metal resistance strategies in the same environment. Thirteen MAGs were selected as representatives of the most abundant populations in the deep layer, where toxic metal concentrations are highest (Table 5). Among these 13 MAGs, the most abundant was MAG 1 (*Euryarchaeota*, order *Thermoplasmatales)* with a relative abundance of 10.3%. The other 12 MAGs had relative abundances between 2% and 0.2%. Representatives of the majority of these populations were also retrieved in our analysis of 16S rRNA reconstructed sequences from metagenomes with EMIRGE (Figure 1). MAGs that were not captured by EMIRGE (e.g., MAGs 7, 8, 9, 10, and 12) corresponded to low abundance (<1%) populations, which could explain why their full-length 16S rRNA sequences were not assembled from the metagenomic data. MAGs 8, 12, and 13 were not included in downstream analyses because less than 500 mRNA reads were mapped to their respective contigs (data not shown).

**Figure 5:**
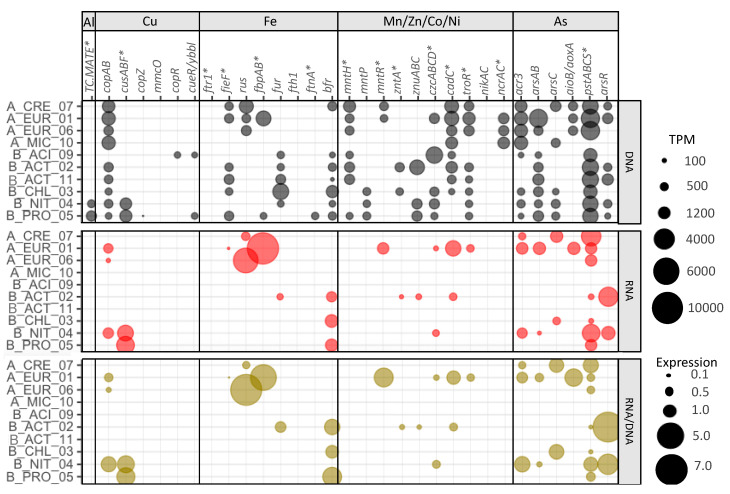
Normalized gene abundance (upper rows), normalized transcript abundance (middle row) and expression (lower rows) of metal-specific metal resistance genes (except with *) detected in MAGs from different phyla recovered from metagenomes of the deep layer of Cueva de la Mora (CM). Normalized gene and transcript abundances are calculated as TPM values. Expression is calculated as transcript TPM/gene TPM. Gene functions are listed in Table 3. MAG labels are clarified in Table 5.

Based on the MRGs annotated in each MAG, these co-existing populations have a wide diversity of strategies for metal resistance (Figure 5). A general feature of the dataset is that the MAG populations have more genetic potential than they expressed at the time of sampling, suggesting the potential to adapt to changing geochemistry and/or metabolic activity levels. All the MAGs except 9 (*Acidobacteriae*) contained *copAB* Cu export genes, but expression was only detected in MAGs 1 and 6 (*Thermoplasmatales*), and 4 (*Thermodesulfovibrio*). MAGs 4 (*Thermodesulfovibrio*) and 5 (*Desulfomonilaceae*) were the only ones with gene-encoded functional potential and expression to export Cu (and/or Ag) by *cusABF*. Not all of the MAGs contained the *fieF* gene (involved in Fe(II) export), and expression was only detected in MAG 1 (*Thermoplasmatales*). The *rus* gene (potentially involved in Fe transport or oxidative stress as stated above) was only found in the *Crenarcheota* MAG 7 (*Nitrososphaerales*) and *Euryarcheota* MAGs (MAGs 1 and 6, both *Thermoplasmatales*). Interestingly, the *rus* gene had higher expression in the less abundant *Thermoplasmatales* MAG 6. The *fbpAB* genes involved in Fe(III) transport were only found and expressed in the MAG 1 (*Thermoplasmatales*). These genes were annotated as part of an Fe(III) transport system by KOFAMscan, but when blasted against the NCBI database and annotated by PFAMscan, they were annotated as a general extracellular-solute binding protein (PF13343.6, SBP_bac_6) not only involved in Fe(III) import but also in the import of multiple oligosaccharides [88]. All bacterial MAGs (2–5, 8, 11, and 13) and MAG 7 (*Nitrososphaerales)* contained the *bfr* gene (involved in Fe storage), but expression was only detected in 3 of the 8 MAGs (Figure 5). The Mn transport regulator gene (*mntR*) was only expressed in MAG 1 (*Thermoplasmatales*). The Co-Zn-Cd efflux system genes (*czcABCD*) were found in many MAGs, but were only expressed in MAG 1 (*Thermoplasmatales*) and 4 (*Thermodesulfovibrio*). None of the MAGs expressed genes involved in transport or efflux of Ni.

All of the MAGs contain genes (*acr3* and/or *arsAB*) that encode for proteins involved in As export, but like the Co-Zn-Cd efflux genes, they were only expressed in MAGs 1 and 4 (Figure 5). Five of the MAGs contained genes that encode for As reducing proteins (*arsC*) and two of the MAGs expressed these genes. However, the most abundant MAGs did not express *arsC*, as we expected considering that speciation modeling predicted As in the deep layer should be essentially 100% reduced (Table 2). MAG 1 expressed a gene annotated as an As(III) oxidase gene (*aioB*/*aoxA* small subunit) that could be involved in electron transport but not in the redox transformation of As since, as explained above, the long subunit of the As(III) oxidase (*aioA*/*aoxB*) was not found in the dataset. Most MAGs contained and expressed the *pstABCS* genes that are involved in both phosphate acquisition and As(V) import. Given that most of the As in the deep layer is in the form of As(III) (Table 2), the *pstABCS* genes might be more important for phosphate acquisition in this environment. MAG 2 (*Thermoleophilia*) did not express genes involved in export or transformation of As, but it has a highly expressed *arsR* that is a regulatory repressor of the *ars* operon [89].

With respect to the potential for synthesis, assembly and transport of exopolysaccharides as a proxy for extracellular sequestration, few of the marker genes were annotated in the MAGs (Figure S3). Only four of the MAGs contained the genes encoding for glycosyltransferases (*exoM*, *wcaL*, *bcsA*, and *gumH*), but only transcripts for *gumH* were found in MAG 2 (*Thermoleophilia)*. The gene encoding for the polysaccharide transporter (*wzx*/*rfbX/gumJ*) was found in most of the MAGs, except for MAG 11 (*Thermoleophilia)* and MAG 4 (*Nitrospirae)*. MAGs 9, 2, 4, and 5 (*Acidobacteriae*, *Thermoleophilia*, *Thermodesulfovibrio*, *Desulfomonilaceae*) contained other genes part of the Wzx-wzy dependent pathway for assembly and transport of exopolysaccharides, consistent with all being Gram-negative bacteria. None of the MAGs contained genes for the ABC-transport pathway.

Our results suggest that metal resistance mechanisms differ between microbial populations in the same environment, including closely related species. For example, the two *Thermoplasmatales* MAGs (1 and 6) had strong similarities in their metal resistance profiles but also important differences, including the Fe(III) importer genes *fbpAB*, the Mn transport regulator genes *mntR*, and the Co/Zn/Cd exporter genes *czcABCD*, all found only in MAG 1. The two *Thermoleophilia* MAGs (2 and 11) also had many similarities, but *zntA* and *znuABC* were found only in MAG 2. These differences are consistent with recent omics-enabled studies documenting significant differences in metabolic pathways and ecological strategies among related species or strains [60,90,91]. While these studies lead us to expect the types of differences among populations that we observed, there are some inherent disadvantages of in silico analysis that could prevent detection of MRGs that are actually present and/or expressed. Plasmids containing metal resistance genes [60] may be under-represented in the genome of a MAG due to their different copy number and sequence composition compared to core genome sequences [92]. Another potential disadvantage is that some reads were not mapped to low-abundance MAGs due to insufficient sequencing depth. Finally, it is extremely likely that existing catalogs of MRGs are incomplete and that a full understanding of metal resistance mechanisms in nature will require targeted studies using a more diverse range of model microorganisms.

## 4. Conclusions

Using metagenomics, we learned that single-celled eukaryotes in the genus *Coccomyxa* dominate the surface layer of Cueva de la Mora (CM), archaea (predominantly *Thermoplasmatales*) dominate the deep layer, and a combination of bacteria and *Coccomyxa* are abundant at the chemocline. Several intriguing patterns emerged from our exploration of metal resistance genes and gene expression in these extreme acidophile communities. First, there were broad differences in metal resistance strategies expressed by eukaryotes, bacteria, and archaea. The most abundant metal resistance transcripts from eukaryotes had putative functions related to import, export, and intracellular accumulation (chelation/storage) of metals. In contrast, the most abundant transcripts from archaea had putative functions related to biochemical transformations. Transcripts from bacteria were more evenly associated with all five metal resistance mechanisms. Second, in contrast to our expectation, expression patterns for metal-specific MRGs did not typically correlate with dissolved metal concentrations or toxicity estimates derived from geochemical modeling (Figure S2). For example, we observed approximately equal expression of As and Cu resistance genes in all three lake layers even though dissolved As and Cu concentrations varied by over two orders of magnitude with depth. Third, metal resistance profiles in co-existing, abundant deep layer populations were diverse even at the species level, consistent with pure culture studies that have shown differences in metal resistance mechanisms at the strain level. Lastly, expression patterns for the biosynthesis and export of EPS showed that microbial populations potentially use EPS for extracellular sequestration of metal(loid)s.

Our observations are based on a manually curated marker gene list of 222 KOs associated with known metal resistance mechanisms. Although abundant in this extremely metal-rich AMD environment, some populations expressed very few of the MRGs known to date, suggesting that additional MRGs remain to be identified. Future work focused on MRGs in uncultivated acidophiles and experiments validating the expression of MRGs under different growth conditions are necessary to further improve our understanding of how acidophiles resist toxic metals at high concentrations. Progress in this direction will expand our ability to engineer bioremediation strategies for acidic pit lakes such as CM and AMD-impacted systems in general.

## Figures and Tables

**Table 1:** Geochemical characteristics of the three layers of Cueva de la Mora (CM) featuring metal(loid)s with known toxicity. Results reflect historical mean values for May as biomass samples were collected in May 2018. All concentrations are in µg/L. Data previously collected by J.S.E, compiled from previous papers by the authors [28,31,32].

	3-m Depth Upper Oxic Layer	11-m Depth Chemocline	35-m Depth Deep Anoxic Layer	In-Stream Standard ^
pH	2.6	3.95	4.5	6.5–9.0
ORP (mV)	575	60	41	not specified
SC (mS/cm)	3.4	4.85	12.1	not specified
T (°C)	17	12.5	18.3	not specified
SO_4_	2,500,000	3,900,000	12,100,000	250,000
Cl	15,000	14,000	22,000	230,000
Al	140,000	158,000	5090	87
As(III)	--	--	17,200	150
As(V)	100	502	0.13	
Co	2010	1310	2040	19
Cu	6010	60	50	9.0
Fe(III)	118,000	--	--	1000
Fe(II)	--	951,000	6,310,000	
Mn	19,000	35,200	116,000	167 *
Ni	443	655	917	52
Zn	13,000	35,200	109,000	120
PO_4_-P	<50	<50	3000	not specified
NH_4_-N	25	400	536	not specified

^ USEPA National Ambient Water Quality Criteria—Constant Contact Concentration Factor. * Mn standard calculated as (Fe standard/6) corresponding to concentration ratio of USEPA drinking water standards. -- refers to species not predicted by geochemical modeling.

**Table 2:** Summary of toxic potency factors and geochemical speciation modeling for the three layers of Cueva de la Mora. Toxicity rankings were based on total element concentration or free cation activity divided by its corresponding USEPA National Ambient Water Quality Criteria constant contact concentration factor (NAWQC-CCCF) numerical standard (Table 1). Speciation results were calculated using PHREEQC for each lake layer and include predominant species % and free cation %.

	3-M Depth Upper Oxic Layer	11-M Depth Chemocline	35-M Depth Deep Anoxic Layer
Toxicity potency factor (TPF-1) ranking based on total concentration	Al > Cu > Fe(III) ~ Mn ~ Zn ~ Co > Ni > As(V)	Al > Fe(II) > Zn > Mn > Co > Ni > Cu > As(V)	Fe(II) >> Zn > Mn > As(III) ~ Co > Al > Ni > Cu
Toxicity potency factor (TPF-2) ranking based on free cation activity *	Cu > Al > Mn > Co > Zn > Fe(III) ~ Ni	Fe(II) > Al > Mn > Zn > Co > Ni > Cu	Fe(II) >> Mn > Zn > Co > Ni > Al > Cu
**Al**	TPF-1 = 1600;	TPF-1 = 1800;	TPF-1 = 60;
	TPF-2 = 190	TPF-2 = 230	TPF-2 = 4.0
	AlSO_4_^+^ = 83%;	AlSO_4_^+^ = 78%;	AlSO_4_^+^ = 72%;
	Al^3+^ = 12%	Al^3+^ = 13%	Al^3+^ = 6%
**As(V)**	TPF-1 = 0.7;	TPF-1 = 3.3;	--
	TPF-2 = n.a.	TPF-2 = n.a.	
	H_2_AsO_4_^–^ = 89%	H_2_AsO_4_^–^ = 9%	
**As(III)**	--	--	TPF-1 = 110;
			TPF-2 = n.a.
			H_3_AsO_3_ = 100%
**Co**	TPF-1 = 106;	TPF-1 = 69;	TPF-1 = 107;
	TPF-2 = 78	TPF-2 = 50	TPF-2 = 85
	Co^2+^ = 74%;	Co^2+^ = 73%;	Co^2+^ = 80%;
	CoSO_4_ = 26%	CoSO_4_ = 27%	CoSO_4_ = 20%
**Cu**	TPF-1 = 670;	TPF-1 = 6.7;	TPF-1 = 5.6;
	TPF-2 = 440	TPF-2 = 3.1	TPF-2 = 2.5
	Cu^2+^ = 66%;	Cu^2+^ = 47%;	Cu^2+^ = 45%;
	CuSO_4_ = 34%	CuSO_4_ = 53%	CuSO_4_ = 55%
**Fe(III)**	TPF-1 = 120;	--	--
	TPF-2 = 6.0		
	FeSO_4_^+^ = 51.0%;		
	Fe^3+^ = 5.0%		
**Fe(II)**	--	TPF-1 = 950;	TPF-1 = 6300;
		TPF-2 = 660	TPF-2 = 4800
		Fe^2+^ = 69.1%;	Fe^2+^ = 76.4%;
		FeSO_4_ = 30.5%	FeSO_4_ = 23.6%
**Mn**	TPF-1 = 110;	TPF-1 = 210;	TPF-1 = 700;
	TPF-2 = 90	TPF-2 = 160	TPF-2 = 570
	Mn^2+^ = 76.2%;	Mn^2+^ = 76.0%;	Mn^2+^ = 81.8%;
	MnSO_4_ = 23.4%	MnSO_4_ = 23.9%	MnSO_4_ = 18.2%
**Ni**	TPF-1 = 9.0;	TPF-1 = 13;	TPF-1 = 18;
	TPF-2 = 6.0	TPF-2 = 5.0	TPF-2 = 8.0
	Ni^2+^ = 68.6%;	NiSO_4_ = 62.2%;	NiSO_4_ = 54.9%;
	NiSO_4_ = 31.1%	Ni^2+^ = 37.5%	Ni^2+^ = 44.3%
**Zn**	TPF-1 = 110;	TPF-1 = 290;	TPF-1 = 910;
	TPF-2 = 70	TPF-2 = 150	TPF-2 = 130
	Zn^2+^ = 63.9%;	Zn^2+^ = 52.7%;	Zn(SO_4_)_2_^2−^ = 67.0%;
	ZnSO_4_ = 31.7%	ZnSO_4_ = 34.6%	Zn^2+^ = 14.0%

* Arsenic has no TPF-2 values because it is not cationic (n.a. = not assigned). -- refers to species not predicted by the geochemical modeling.

**Table 3:** Description of key metal resistance genes examined in Cueva de la Mora (CM).

Metal	Name	Description-Function	Mechanism	KO Identifier
Al	* TC.MATE	Multidrug resistance protein, part of the multidrug and toxin extrusion (MATE) family, Al and other drugs tolerance, also known as *SLC47A*, *mtdK*, *dinF* ^b^	Export	K03327
Cu	*copAB*	P-type Cu+ transporters, also involved in resistance to sodium acetate and Ag in certain organisms ^a^, catalyze the translocation of inorganic cations ^b^, *copA* also known as *ctpA* and *ATP7* ^b^, *copB* also known as *copA_3*, *copF_3*, *cadA ^b^*	Export	K17686, K01533
	*cueR/ybbI*	MerR family transcriptional regulator, Cu efflux regulator, also involved in resistance to hydrochloric acid (HCl) ^a^	Regulation	K19591
	*mmcO*	Multicopper oxidase, oxidize metal ions (Cu) with oxygen as acceptor, also known as *copA_1*, *copA_2*, *cueO* ^b^	Bioch Trans	K22552
	*copR*	Two-component system, OmpR family, copper resistance phosphate regulon response regulator CusR ^b^	Regulation	K07665
	* cusABF	Cu(I)/Ag(I) efflux system membrane proteins, also known as *silABF* ^b^	Export	K07787, K07798, K07810
	*copZ*	Copper chaperone ^ab^	Export	K07213
Fe	* ftr1	High affinity low-pH Fe(II) transporter ^b^, also involved in Pb resistance ^a^	Import	K07243
	* fieF	Ferrous-iron efflux pump ^b^, also involved in efflux of Zn/Co/Cd/Ni ^a^	Export	K13283
	* fbpAB	Iron(III) transport system substrate-binding protein, ABC transporters, also known as *afuAB* ^b^, also involved in Ga resistance ^a^	Import	K02012, K02011
	* rus	Rusticyanin, involved in Fe(II) oxidation, but potentially in copper resistance [68]	Bioch Trans	K18683
	*fur*	Fur family transcriptional regulator, ferric uptake regulator, also known as *zur* and *furB* ^b^	Regulation	K03711
	*fth1*	Ferritin heavy chain, iron storage mainly found in eukaryotes ^b^	Int Accu	K00522
	* ftnA	Ferritin, iron storage, also involved in resistance to Cu and Mn ^a^.	Int Accu	K02217
	*bfr*	Bacterioferritin, iron storage ^b^	Int Accu	K03594
Mn	*mntP*	Manganese efflux protein ^ab^	Export	K23242
	* mntH	Manganese transport protein involved in Mn, Zn, and Fe uptake ^a^	Import	K03322
	* mntR	DtxR family transcriptional regulator, manganese transport regulator ^b^, responds to Mn(II), Fe(II), Zn(II), Cd(II), Co(II) [69]	Regulation	K11924
Zn	* zntA	Zn2 + /Cd2 + -exporting ATPase ^b^, also involved in Co and Pb extrusion ^a^	Export	K01534
	*znuABC*	Zinc transport system ^b^	Import	K09815, K09816, K09817
	* czcABCD	Cobalt-zinc-cadmium efflux system ^b^, also involved in Ni and Co resistance ^a^	Export	K15726, K15727, K15725, K16264
	* cadC	ArsR family transcriptional regulator, responsive transcriptional repressor ^b^, involved in Cd/Bi/Zn/Pb resistance ^a^	Regulation	K21903
	* troR	Mn-dependent transcriptional regulator ^b^, involved in resistance to Zn/Mn/Fe and Hydrogen Peroxide ^a^	Regulation	K03709
Ni	*nikAC*	Nickel transport system ^b^	Import	K15584, K15586
	* ncrAC	Ni/Co transporters, involved also in Co/Cd/Zn/Fe, also known as *nrsD/rcnA/yohM* ^ab^	Export	K07785, K08970
As	*acr3*	Arsenite transporter ^b^	Export	K03325
	*arsAB*	Arsenite/tail-anchored protein-transporting ATPase and pump membrane protein ^b^	Export	K01551, K03893
	*arsC*	Arsenate reductase (thioredoxin as acceptor) ^b^	Bioch Trans	K03741
	*arsC_HAC1*	Arsenate reductase (glutathione or glutaredoxin as acceptor) ^b^	Bioch Trans	K22547
	*aioB/aoxA*	Arsenite oxidase small subunit ^b^	Bioch Trans	K08355
	* pstABCS	Phosphate transport system, As(V) uptake ^a^	Bioch Trans	K02038, K02036, K02037, K02040
	*arsR*	Transcriptional repressor, As resistance, regulation ^b^	Regulation	K03892
	*acr1*	AP-1-like transcription factor, As resistance, regulation ^b^	Regulation	K09043

^a^ Information taken from BacMet database. ^b^ Information taken from KEGG database. ^c^ Mechanisms = Biochemical Transformation (Bioch Trans), Regulation, Extracellular sequestration (Ext Seq), Intracellular accumulation (Int Acc). * Non-specific metal gene.

**Table 4:** Description of marker genes involved in the synthesis, assembly, and transport of extracellular and capsular polysaccharide substances [20,58,84] that are displayed in Figure 4. All genes used as a proxy for extracellular metal sequestration are included in Table S1.

Gene	Description-Function	Pathway	KO Identifier
***exoQ***	Exopolysaccharide production protein ExoQ (polymerase wzy)	Wzx-Wzy dependent pathway	K16567
***wzxC***	wzxC; lipopolysaccharide exporter (flippase wzx)	Wzx-Wzy dependent pathway	K16695
***wzx*** **/*rfbX*/*gumJ***	Polysaccharide transporter, PST family (flippase wzx)	Wzx-Wzy dependent pathway	K03328
***wzc***	Tyrosine-protein kinase Etk/Wzc (polysaccharide co-polymerase PCP)	Wzx-Wzy dependent pathway	K16692
***epsB***	Protein-tyrosine kinase (polysaccharide co-polymerase PCP)	Wzx-Wzy dependent pathway	K00903
***exoP***	Polysaccharide biosynthesis transport protein (polysaccharide co-polymerase PCP)	Wzx-Wzy dependent pathway	K16554
***gumC***	GumC protein (polysaccharide co-polymerase PCP)	Wzx-Wzy dependent pathway	K13661
***wza***	Polysaccharide biosynthesis/export protein (outer membrane transporter OPX)	Wzx-Wzy dependent pathway	K01991
***kpsT***	Capsular polysaccharide transport system ATP-binding protein (ABC-transporter)	ABC-transport	K09689
***kpsM***	Capsular polysaccharide transport system permease protein (ABC-transporter)	ABC-transport	K09688
***kpsE***	Capsular polysaccharide transport system permease protein (polysaccharide co-polymerase PCP)	ABC-transport	K10107
***exoA***	Succinoglycan biosynthesis protein ExoA	Glycosyltransferase	K16557
***exoM***	Succinoglycan biosynthesis protein ExoM	Glycosyltransferase	K16556
***wcaL***	Colanic acid/amylovoran biosynthesis glycosyltransferase	Glycosyltransferase	K16703
***gumH***	Alpha-1,3-mannosyltransferase	Glycosyltransferase	K13657
***bcsA***	BcsA; cellulose synthase (UDP-forming)	Glycosyltransferase	K00694

**Table 5:** Metagenome-assembled genomes (MAGs) obtained from the deep layer (35-m) of Cueva de la Mora (CM) representing different phyla (see Figure 1). MAG labels are constructed to indicate Domain_Phylum_Abundance Rank. Relative abundances (Rel. Abu.) were calculated based on total DNA-reads mapped to each MAG. Completeness (Com) and Contamination (Con) percentage as calculated by CheckM [86] are also included. All MAGs were of medium quality based on [87]. All MAGs have <96.5% average nucleotide identity (ANI).

MAG	Taxonomy Based on the Genome Taxonomy Database (GTDB)	Rel. Abu. (%)	Com (%)	Con (%)
A_CRE_07	*d__Archaea;p__Crenarchaeota;c__Nitrososphaeria;o__Nitrososphaerales;f__UBA183;g__UBA183*	0.7	95	5
A_EUR_01	*d__Archaea;p__Thermoplasmatota;c__Thermoplasmata;o__Thermoplasmatales;f__GCA-001856825;g__GCA-001856825*	10.3	94	3
A_EUR_06	*d__Archaea;p__Thermoplasmatota;c__Thermoplasmata;o__Thermoplasmatales;f__Thermoplasmataceae*	1.2	94	1
A_MIC_10	*d__Archaea;p__Micrarchaeota;c__Micrarchaeia;o__Micrarchaeales;f__Micrarchaeaceae;g__UBA12276*	0.5	77	0
A_NAN_12	*d__Archaea;p__Nanoarchaeota;c__Nanoarchaeia;o__Woesearchaeales;f__UBA525;g__UBA153*	0.4	77	0
B_ACI_09	*d__Bacteria;p__Acidobacteriota;c__Acidobacteriae*	0.5	81	4
B_ACT_02	*d__Bacteria;p__Actinobacteriota;c__Thermoleophilia;o__BMS3ABIN01;f__BMS3ABIN01*	2.8	87	1
B_ACT_11	*d__Bacteria;p__Actinobacteriota;c__Thermoleophilia;o__BMS3ABIN01;f__BMS3ABIN01;*	0.4	90	3
B_CHL_03	*d__Bacteria;p__Chloroflexota;c__Dehalococcoidia;o__SZUA-161;f__SZUA-161*	2.3	98	2
B_DOR_08	*d__Bacteria;p__Dormibacterota;c__Dormibacteria;o__UBA8260;f__UBA8260*	0.7	88	0
B_NIT_04	*d__Bacteria;p__Nitrospirota;c__Thermodesulfovibrionia;o__Thermodesulfovibrionales;f__JdFR-88*	2.3	100	1
B_PAT_13	*d__Bacteria;p__Patescibacteria;c__Paceibacteria;o__UBA6257;f__Colwellbacteraceae*	0.2	75	0
B_PRO_05	*d__Bacteria;p__Desulfobacterota;c__Desulfomonilia;o__Desulfomonilales;f__Desulfomonilaceae*	1.3	92	1

## Supplementary Materials

The following are available online at https://www.mdpi.com/2076-2607/8/9/1350/s1, Figure S1. Geochemical conditions recorded in Cueva de la Mora acidic pit lake over the last 10 years. Sharp chemocline present ca. 10-m below lake surface. Data previously collected by J.S.E, compiled from previous papers by the authors. Figure S2. Expression (RNA_TPM/DNA_TPM) of specific marker KOs involved in metal resistance versus metal concentration. Figure S3. Normalized gene abundance (upper rows), normalized transcript abundance (middle row), and expression (lower rows) of extracellular sequestration marker genes detected in the co-assembled metagenomes from the three layers of Cueva de la Mora. Normalized gene and transcript abundances are calculated as TPM values. Expression is calculated as transcript TPM/gene TPM. All of these genes are involved in biosynthesis of polysaccharides (Glycosyltransferases), and polysaccharide assembly and transport (Wzx-wzy dependent pathway and ABC transport). 1 and 2 refers to replicates (DNA or mRNA reads from each replicate mapped to co-assembled metagenomes). Gene functions and their respective KOs are listed in Table 4. mRNA reads from CM35_2 are not included due to low mapping rates to the co-assembled metagenome. Table S1. Metal resistance marker genes list with its respective KO, BacMet ID (if found), mechanisms and metal related. Table S2. General statistics of metagenomes and metatranscriptomes. Table S3. General statistics and taxonomic classification of the 16S and 18S rRNA reconstructed with EMIRGE from metagenomes of the upper layer (CM03), chemocline (CM11), and deep layer (CM35). Table S4. Predicted genes annotated as metal resistance genes in the co-assembled metagenomes of the three layers and their respective TPM values from metagenome and metatranscriptome replicates.
